# Clinical Relevance of Ratios Derived from Routine Blood-Based Biomarkers in Cholangiocarcinoma: A Retrospective Cohort Study

**DOI:** 10.3390/medicina61122166

**Published:** 2025-12-05

**Authors:** Amalia Debora Ventuneac, Andrada Seicean, Nadim Al Hajjar, Romeo Chira, Andra Ciocan, Vlad Andrei Ichim, Sorana D. Bolboacă

**Affiliations:** 1 Department of Medical Informatics and Biostatistics, Faculty of Medicine, “Iuliu Hațieganu” University of Medicine and Pharmacy, 6 Pasteur Street, 400349 Cluj-Napoca, Romania; amalia.debo.ventuneac@elearn.umfcluj.ro; 2 Department of Internal Medicine, Medical Clinic I, Faculty of Medicine, “Iuliu Hațieganu” University of Medicine and Pharmacy 3-5 Clinicilor Street, 400006 Cluj-Napoca, Romania; ioan.chira@umfcluj.ro (R.C.); ichim.vlad@umfcluj.ro (V.A.I.); 3 Department of Internal Medicine, Medical Clinic III, Faculty of Medicine, “Iuliu Hațieganu” University of Medicine and Pharmacy, 19-21 Croitorilor Street, 400162 Cluj-Napoca, Romania; andrada.seicean@umfcluj.ro; 4 “Octavian Fodor” Regional Institute of Gastroenterology and Hepatology, 19-21 Croitorilor Street, 400162 Cluj-Napoca, Romania; nadim.alhajjar@umfcluj.ro; 5 Department of Surgery—Surgery III, Faculty of Medicine, “Iuliu Hațieganu” University of Medicine and Pharmacy Cluj-Napoca, 19-21 Croitorilor Street, 400162 Cluj-Napoca, Romania; 6 Medical Clinic 1, Cluj Emergency County Hospital, 3-5 Clinicilor Street, 400006 Cluj-Napoca, Romania

**Keywords:** cholangiocarcinoma (CCA), lymphocytes (LYM), inflammation, biomarker

## Abstract

*Background and Objectives:* Cholangiocarcinoma (CCA) is an aggressive tumor that originates in the biliary tract and is subdivided anatomically into intrahepatic (iCCA) and extrahepatic (perihilar-pCCA and distal-dCCA). Diagnosis remains challenging, particularly for extrahepatic forms (pCCA and dCCA). We aimed to assess the relation between systemic inflammatory markers—specifically lymphocyte-related ratios—and tumor characteristics in a Romanian cholangiocarcinoma cohort. *Materials and Methods:* We conducted an exploratory single-center study including adult patients with a confirmed CCA histopathological diagnosis. We excluded patients with an uncertain diagnosis or tumors of the ampulla of Vater or gallbladder. Demographic and clinical data were retrospectively collected from medical records. *Results:* Tumor localization was the strongest predictor of metastatic disease. The odd of metastasis was 7.3 times higher for iCCA than dCCA and 4.5 times higher for iCCA than pCCA. Although several evaluated inflammatory biomarkers showed statistically significant associations, their clinical relevance was limited. The odds ratios for these biomarkers were characterized by lower bounds near the null value and wide confidence intervals, reflecting considerable patient heterogeneity, model instability, and inconclusive effect sizes. *Conclusions:* Our findings suggest a potential biological link between systemic inflammation, metastatic spread, and tumor differentiation grade that deserves further investigation using more accurate systemic inflammation biomarkers than those routinely collected.

## 1. Introduction

Cholangiocarcinoma (CCA) is a tumor that originates in the biliary tract and can be subdivided anatomically into two main types: intrahepatic (iCCA), which develops between the bile ductules and the bile ducts of second order, and extrahepatic (eCCA), which arises distally of those mentioned above. The eCCA can be categorized as perihilar (pCCA), which emerges in the left and/or right hepatic duct and/or at their confluence and distal cholangiocarcinoma (dCCA), which involves the main bile duct. The iCCA is considered the second most prevalent primary hepatic tumor, after hepatocellular carcinoma. As treatment options differ, a distinction between pCCA and dCCA must be made [1,2]. Cholangiocarcinoma is considered a rare cancer in most parts of the world, with an incidence of less than 6 cases per 100,000 people. However, incidence on the Asian continent is higher, with reports of up to 85 cases per 100,000 inhabitants in Thailand [3], where *Opisthorchis viverrini*, a liver fluke associated with bile duct inflammation is endemic [4]. Cholangiocarcinomas are aggressive tumors, with a median overall survival from diagnosis that ranges from 16.1 to 18 months, depending on whether or not there is a possibility to perform surgery with curative intent [5,6], but between 48.5% and 55% of iCCA patients undergoing surgery associated with adequate lymphadenectomy (retrieval of ≥6 lymph nodes) have metastatic lymph nodes [7]. Median overall survival in patients with biliary tract cancers (including gallbladder cancer) in a metastatic state is 4.5 months [8]. Mortality rates for iCCA per 100,000 person-years are higher than mortality rates for eCCA in Europe, with Ireland having the highest recorded rates of 2.67/100,000 person-years for the intrahepatic forms. Moreover, mortality trends are presenting an ascending tendency for iCCA in most European countries, with the highest average annual percentage change (AAPC) of 18.87% recorded in the Lithuanian population [9].

Multiple risk factors have been linked to CCA, some of the most notable are choledochal cysts with an odd ratio (OR) of 26.71 (95% confidence interval (CI) 15.8–45.16) for the development of iCCA and an OR of 34.49 (95% CI 24.36–50.12) for eCCA [10], Caroli disease and primary sclerosing cholangitis (PSC) [11].Infection with liver flukes such as *Opisthorchis viverrini,* more characteristic for the Asian population, presents an OR of 6.35 (95% CI: 2.87–14.05) for the development of CCA, irrespective of its anatomical location [12]. History of choledocholithiasis is considered a risk factor for cholangiocarcinoma, with 2.64% of patients being diagnosed with CCA during a 10-year follow-up from the moment choledochal lithiasis was diagnosed [13]. Cholelithiasis, cirrhosis, infection with hepatitis B (HBV) and C viruses (HCV) have also been mentioned as risk factors for CCA, alongside other more common diseases such as type 2 diabetes (T2D), obesity, hypertension, and habits such as smoking or alcohol intake [10,11]. The presence of either systemic or local inflammation is the common point of some of the above-mentioned risk factors, which also leads to the assumption that inflammation itself could be a risk factor for tumorigenesis and, in turn, for the development of CCA and for the distant dissemination of tumoral tissue with several types of cytokines, chemokines, and growth factors playing a role in this process [14,15,16].

The diagnostic approach in patients with pCCA or dCCA, as per the latest European Association for the Study of the Liver (EASL) guidelines, includes a definitive histopathological diagnosis, in all cases deemed feasible, regardless of the initial appreciation of the tumor’s resectability. Current recommendations consider the possibility of a multidisciplinary tumor-board-based diagnosis in cases of patients under suspicion for eCCA with no histopathological confirmation. In patients with intrahepatic forms of cholangiocarcinoma, the diagnosis is also based on histopathological confirmation [2]. Imagistic staging should include both magnetic resonance cholangiopancreatography (MRCP) for local assessment and computed tomographic (CT) for evaluation of systemic disease in cases of iCCA and eCCA. Tumor markers are not routinely recommended to support the diagnosis of cholangiocarcinoma, but bear a negative prognostic value [2,17].

Information regarding Romanian cholangiocarcinoma patients is scarce, and the latest article available dates to 2021. Prognosis is reduced in this population, with a 2-year survival rate of up to 5.5% for dCCA [18]. Diagnostic sensitivity of ultrasound for all three subtypes of cholangiocarcinoma was 73.5% in Romanian patients. The lowest sensitivity was recorded for patients with dCCA, with 33.3% [19]. One of the main issues in the management of patients with cholangiocarcinoma was the difficulty of obtaining an accurate histopathological diagnosis, which aligns with challenges described in other populations [20,21].

We aimed to evaluate the lymphocyte-related ratios in patients with cholangiocarcinoma, taking into account the presence of metastases at diagnosis and tumor localization. Secondly, an exploratory analysis of lymphocyte-related ratios according to tumor differentiation grade as outcome was also conducted.

## 2. Materials and Methods

The study was conducted following the Declaration of Helsinki and has been approved by the Ethics Committee of the “Iuliu Haţieganu” University of Medicine and Pharmacy Cluj-Napoca (No. 150/10.06.2025) and by the Ethics Committee of the “Professor Doctor Octavian Fodor” Regional Institute of Gastroenterology-Hepatology (No. 7202/17.06.2025). The ethics committee waived the requirement for informed consent as the study involved only the analysis of pre-existing, routinely collected healthcare data.

### 2.1. Study Design

Our study is conducted using routinely collected health data on patients with definitive histopathological diagnosis of cholangiocarcinoma who received medical treatment at the “Professor Doctor Octavian Fodor” Regional Institute of Gastroenterology-Hepatology.

We included in the sample adult patients (≥18 years of age) with a histopathological diagnosis of cholangiocarcinoma, irrespective of the anatomical location, with complete white blood count (WBCs) and platelet data available in the electronic hospital system. We targeted patients who were hospitalized between 1 January 2018, and 31 December 2022, regardless of the year of diagnosis. Patients with tumors of the gallbladder or the ampulla of Vater, those with inconclusive biopsy results, or incomplete CBC data were excluded.

The applied methodology adheres to the RECORD guideline [22].

### 2.2. Data Collection and Cleaning

The hospital electronic system was the source for data collection. The search was done using the following diagnostic codes included in the International Classification of Diseases, Tenth Revision (ICD-10): C22.1—Intrahepatic bile duct carcinoma, C24.0—Malignant neoplasm of extrahepatic bile duct, C24.8—Malignant neoplasm of overlapping sites of biliary tract, and C24.9—Malignant neoplasm of biliary tract, unspecified.

The following variables were collected retrospectively: demographic data (sex, age at diagnostic, residence), co-morbidities (obesity, Diabetes Mellitus-DM, hypertension), related diseases (cholangitis, cholelithiasis, choledocholithiasis), WBCs (leukocyte-WBC, lymphocytes-LYM, monocytes-MON, and neutrophils-NEU; expressed as 10^9^/L), platelets (PLT), and, whenever available, CRP (C-reactive protein, mg/dL). The following pathology-related variable data were collected: cholangiocarcinoma type (intrahepatic, perihilar, and distal), metastases at diagnosis, and differentiation grade (G1—well differentiated, G2—moderately differentiated, G3—poorly differentiated) whenever available. All collected data were at the time of diagnosis.

The 10^9^/L format was used for all blood cell counts to ensure consistency in units. Data accuracy was verified by checking if quantitative values fall within expected ranges and no contradictions within or between fields (e.g., WBCs are no less than the sum of LYM, MON, and NEU). Verification was conducted when inconsistencies were observed, and data were corrected accordingly. After verification of accuracy, all personally identifiable information (e.g., names, patient IDs, addresses) was removed in compliance with data protection regulations (e.g., GDPR). Missing data were reported as missing and were not subjected to any imputation or handling procedure.

### 2.3. Statistical Analysis

Nine derived variables were created using blood cell counts routinely collected as input data (Table 1).

Statistical analysis was conducted in an exploratory manner. Categorical variables are presented as numbers and percentages and compared using Chi-squared or Fisher’s exact test, as appropriate. The distribution of quantitative variables was tested using the Shapiro–Wilk test for each group and was considered distant from the theoretical normal distribution when *p*-values were less than 0.05. Median and interquartile range (defined as [Q1 to Q3], where Q is the value of the first-Q1 quartile and third-Q3 quartile) were reported when the distribution proved far away from the theoretical normal distribution. Consequently, non-parametric tests were used to compare non-normally distributed data: the Mann–Whitney test for metastasis status as the dependent variable, and the Kruskal–Wallis test for tumor localization and tumor grade.

Logistic regression or ordinal regression analysis was conducted to test the prediction potential of lymphocyte-related ratios and the outcomes of interest whenever statistically significant differences were observed between subgroups. Crude (unadjusted) and adjusted ORs (odd ratios) with associated 95% confidence intervals [lower bound to upper bound] were reported. The overall fit of the regression model was evaluated using the Akaike Information Criterion (AIC), with lower values indicating a better model fit.

All statistical analyses, including descriptive and inferential methods, were conducted using the Jamovi software (v. 2.6.26.0). Figures were generated using Microsoft Excel or Jamovi, and the significance level was set at α = 0.05.

## 3. Results

Three hundred twenty-seven patients, aged between 36 and 89 years, were included in the study. Most participants were male (193, 59.0%), lived in an urban area (211/327, 64.5%), and had hypertension as a comorbidity (175/327, 53.5%). A quarter (82/327, 25.1%) had a diagnosis of diabetes mellitus, 42 (12.8%) were obese, and 27 (8.3%) also had other types of cancer. Eighty-six patients (26.3%) had a medical history of cholelithiasis, and 22 (6.7%) had choledocholithiasis. Fifty-nine patients (18.0%) had cholangitis at the time of diagnosis. One hundred and fifteen patients (35.2%) had metastases at time of the diagnosis. No preponderance of pathology was recorded according to tumor location: 116 (35.5%) were intrahepatic (iCCA), 112 (34.3%) perihilar (pCCA), and 99 (30.3%) distal cholangiocarcinoma (dCCA). Information regarding the differentiation grade was available for 214 patients (65.4%), with a preponderance of moderately differentiated tumors (97, 45.3%) and poorly differentiated (64, 29.9%), opposite to well differentiated (53, 24.8%).

The age at diagnosis was statistically significant different (Mann–Whitney test: *p* = 0.027), when men (median = 66 years, IQR = [61 to 71]) were compared to women (median = 64.5 years, IQR = [56 to 70]). A higher percentage of women had metastases at diagnosis (54/134, 40.3%) compared to men (61/193, 31.6%), the difference being statistically significant (Chi-squared test: χ^2^ = 2.62, *p*-value = 0.105). Localization of tumor was uniform among men (68, 35.2% iCCA, 58, 30.1% pCCA, and 67, 34.7% dCCA), with most of the cases as pCCA (54, 40.3%) and iCCA (48, 35.8%), and less frequent dCCA (32, 23.9%) among women (Chi-squared test: χ^2^ = 5.50, *p* = 0.064). The distribution of differentiation grade was similar among men and women (Chi-squared test: χ^2^ = 0.52, *p*-value = 0.768, n = 214). The values of LMR were higher in women (3.1 [2.3 to 4.5]) than in men (2.3 [1.6 to 3.3]), with statistically significant differences (Mann–Whitney test: *p*-value < 0.001). Men had higher values for N/LPR (1.5 [1 to 2.2]), MWR (7.6 [6.4 to 9.4]), and SIRI (2.5 [1.5 to 4.5]) than women (N/LPR: 1.2 [0.8 to 2]), MWR: 6.4 [4.9 to 8.1], and SIRI: 1.8 [1.2 to 3.3]), the differences being statistically significant (Mann–Whitney test: *p*-value = 0.027 for N/LPR, *p*-value < 0.001 for MWR, and *p*-value = 0.005 for SIRI).

### 3.1. Lymphocyte-Related Biomarkers of Metastases

The age of patients with metastases (65 years [58 to 68]) and those without (66 years [60 to 72]) at diagnosis, either at the lymph node or organ level, was similar (Mann–Whitney test: *p*-value = 0.109). Patients with metastases had more frequent intrahepatic cholangiocarcinoma and higher values of lymphocyte-related biomarkers than those without metastases, except for MWR, which exhibits lower values (Table 2). Only SIRI had similar values regardless of the presence or absence of metastases at diagnosis (Table 2).

The association between CCA type and sex had a tendency towards statistical significance (Chi-squared test: χ^2^ = 5.5, *p*-value = 0.064, n = 327), while statistically significant differences were observed regarding the age between men and women. Considering these observations, these two factors were considered covariates in logistic regression analysis. The risk of metastases was 7.3 times higher for patients with intrahepatic tumors, and 4.5 times higher for patients with perihilar localization compared to those with distal CCA (Table 3). Although lymphocyte-related biomarkers showed potential, the ORs around the value of one (Table 3) indicate limited clinical performance.

### 3.2. Lymphocyte-Related Biomarkers of Cholangiocarcinoma Localization

**Figure 1:**
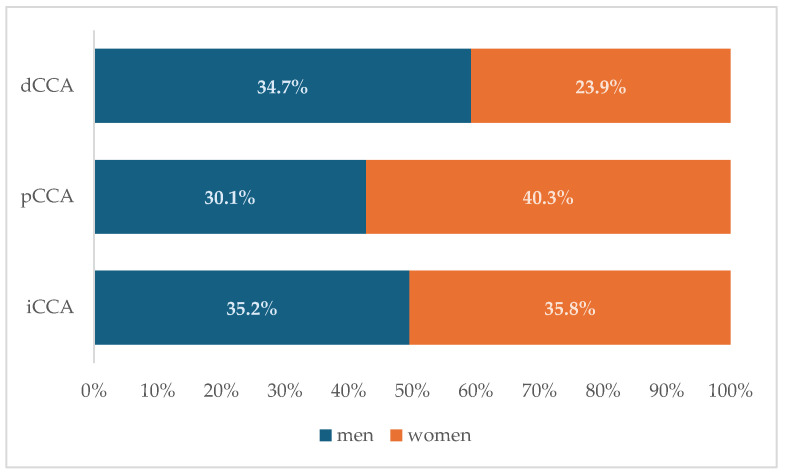
Distribution of cholangiocarcinoma subtypes by sex. (dCCA—distal cholangiocarcinoma; pCCA—perihilar cholangiocarcinoma; iCCA—intrahepatic cholangiocarcinoma).

**Figure 2:**
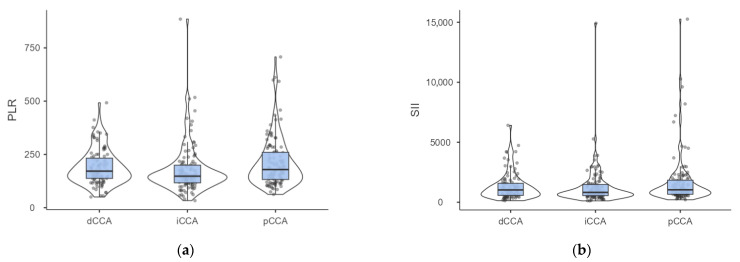
(**a**) Distribution of PLR by cholangiocarcinoma localization. (Dwass-Steel-Critchlow-Fligner pairwise comparisons: *p*-value = 0.011 for iCCA vs. pCCA, *p*-value = 0.067 for iCCA vs. dCCA, *p*-value = 0.752 for pCCA vs. dCCA). (**b**) Distribution of SII by cholangiocarcinoma localization. (Dwass-Steel-Critchlow-Fligner pairwise comparisons: *p*-value = 0.05 for iCCA vs. pCCA, *p*-value = 0.433 for iCCA vs. dCCA, *p*-value = 0.759 for pCCA vs. dCCA; iCCA—intrahepatic; pCCA—perihilar; dCCA—distal cholangiocarcinoma).

No preponderant localization was observed in our cohort (Figure 1). Similar patient characteristics were observed regardless of the localization (Table 4), but with a higher percentage of patients with cholelithiasis and higher values of PLR (Figure 2a) and SII (Figure 2b) among those with perihilar cholangiocarcinoma.

Post hoc analysis of PLR showed statistically significant differences between patients with intrahepatic cholangiocarcinoma and those with perihilar localization (Figure 2a). Similarly, the tendency towards statistical significance observed for SII (Table 4) was also observed in comparison of iCCA with pCCA (Figure 2b).

### 3.3. Lymphocyte-Related Biomarkers and Differentiation Grade of Cholangiocarcinoma

**Figure 3:**
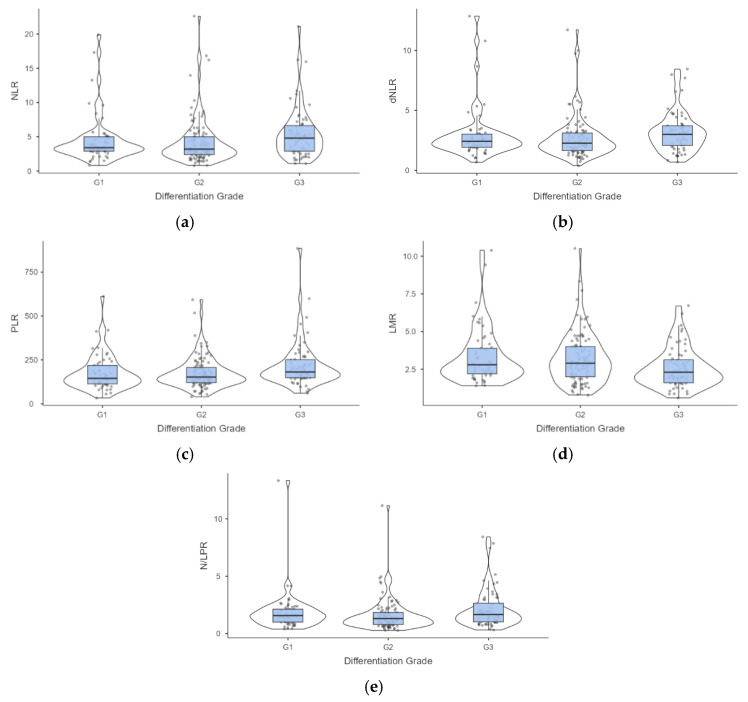
Distribution of values associated with evaluated lymphocyte-related biomarkers by differentiation grade of the cholangiocarcinoma. Post hoc analysis (**a**) NLR—neutrophil-to-lymphocyte ratio: *p* = 0.020 G2 vs. G3; (**b**) dNLR—derived neutrophil-to-lymphocyte ratio: *p* = 0.033 G2 vs. G3; (**c**) PLR—platelet-to-lymphocyte ratio: *p* = 0.036 G1 vs. G3, and *p* = 0.021 G2 vs. G3; (**d**) LMR—lymphocyte-to-monocytes ratio: *p* = 0.025 G1 vs. G3, and *p* = 0.035 G2 vs. G3; (**e**) N/LPR—neutrophil-to-lymphocyte and platelet ratio: *p* = 0.030 G2 vs. G3.

The information regarding the differentiation grade was available for 214 patients aged from 36 to 89 years old, half of them older than 65 years old. Most intrahepatic tumors are moderately or poorly differentiated, and at least half of patients with poorly differentiated tumors had metastases at diagnosis (Table 5). The values of NLR, dNLR, PLR, LMR, and N/LPR proved statistically significant differences by differentiation grade (Table 5), reflected by significant differences between G1 and G3 (Figure 3).

The presence of metastases at the time of diagnosis was a statistically significant predictor for differentiation grade, with the odds of having a poorly differentiated tumor 2.5 times higher for patients with metastases at diagnosis. Similarly, perihilar and distal localization were significant predictors, indicating a lower odd of a poorly differentiated tumor (Table 6). Although PLR and LMR proved statistically significant, supporting their potential as predictors of differentiation grade, they had limited clinical relevance since the ORs values are around the value of one (Table 6).

## 4. Discussion

### 4.1. Main Findings

The risk of metastases in our cohort was higher for patients with intrahepatic (7.4) or perihilar (4.5) tumors compared to those with distal CCA. The values of PLR proved statistically significant for different tumor localizations (*p*-value = 0.009), with SII showing a tendency towards statistical significance (*p*-value = 0.065), as reflected in the comparison of intrahepatic and perihilar localization. Although lymphocyte-related biomarkers showed potential in the differentiation of CCA patients with and without metastases, and the patients with different differentiation grades, the unadjusted and adjusted ORs around the value of one indicate limited clinical relevance. Our findings provide a critical negative result that underscores the challenges of applying readily available lymphocyte-related ratios calculated from routinely measured biomarkers in patients with CCA, highlighting the potential for population-specific variations. Our negative results are of substantial clinical and scientific importance, suggesting that the pursuit of these biomarkers as standalone evaluation tools in CCA may be unproductive.

### 4.2. Findings Interpretation

Distribution of cholangiocarcinoma cases according to anatomical positioning in our cohort shows that all three anatomical subtypes exhibit similar incidence rates. In contrast with our findings, previous epidemiological data on European patients suggest that the most common subtype is pCCA, followed closely by dCCA, with iCCA less frequently [23]. This difference could be explained by our applied inclusion criteria, with evaluation only of the patients with a definitive histopathological diagnosis, while difficulties in tissue sampling have been reported. Sensitivity of 49% and specificity of 96.33% were reported for endoscopic retrograde cholangiopancreatography (ERCP) with the acquisition of either brush cytology or forceps biopsy, and a sensitivity and specificity of 75% and 100%, respectively, was reported for endoscopic ultrasound guided fine-needle biopsies (EUS-FNA) [24]. Another plausible explanation could be the miscoding to the previous use of ICD-10, which did not include a specific diagnostic code for pCCA [25]. In a large retrospective study on the Thai population, only 21.6% of male and 23.1% of female patients with cholangiocarcinoma had a histopathological confirmation of the disease [26]. This finding underscores the technical challenges often encountered in obtaining adequate biopsy samples in this disease.

Our cohort reflected the well-documented male predominance in cholangiocarcinoma [27], with a male-to-female ratio of 1.44:1. Our finding shows that men are significantly older at diagnosis than women. Sex-related differences in patients with cholangiocarcinoma have been previously reported, but men had lower reported age medians at diagnosis than females (66 years vs. 68 years) in the Thai population [26]. Cholangiocarcinoma patients from the United States are similar to our results, with older males than females at diagnosis (mean age of 68.8 ± 0.14 years for men and 67.8 ± 0.14 years for women) [28]. Another sex-related difference in this cohort was that females were more likely to present with metastatic disease at any site (lymph nodes or other organs) at the time of diagnosis. Opposite to our results, male sex has previously been associated with a higher risk for lymph node metastases in patients with iCCA [29]. In our cohort, we observed that perihilar cholangiocarcinoma was significantly more common in women than in men. A higher incidence of iCCA and eCCA in men than in women was reported in American patients, but if stratified according to age, patients older than 80 years were predominantly female [30].

Patients with iCCA and pCCA in our cohort were diagnosed in a more advanced stage than those with dCCA (see Table 2). The presence of metastatic disease was previously reported as more commonly found in iCCA (43.5%), followed by pCCA (30.3%) and dCCA (30.1%) [31], results that partially align with the results of our study. This is an effect of the more clinically evident presentation of patients with eCCA, who are more prone to developing obstructive jaundice due to biliary obstruction. Patients with pCCA present with jaundice (the median of bilirubin of 3.3 mg/dL, IQR = [0.9 to 10.6]) compared to patients with iCCA (0.6 mg/dL, IQR = [0.4 to 1.1]) [32].

Modified systemic inflammation scores are linked to the presence of metastatic disease (Table 2 and Table 3), but are also linked to a poorer degree of tumor differentiation (Table 5, Figure 2). Systemic inflammation scores such as SII or NLR have been associated with the presence of metastases at the time of diagnosis and have been previously reported for prognostic evaluation of cholangiocarcinoma patients [33]. Also, PLR was an independent risk factor for lymph node metastases with OR = 5.14 (95% CI [1.21 to 21.7]), thus being suggested as a valuable tool for pre-surgical assessment of the presence of metastatic lymph nodes [34]. However, inflammation scores, such as SII, did not have any association with the degree of tumoral differentiation [35], a result also observed in our cohort (Table 5).

Patients with metastases were more likely to have a poorly differentiated tumor (Table 5). Previous evidence showed that poorly differentiated tumors are more likely to have lymph node metastases [29].

Logistic regression revealed that tumor localization was significantly associated with metastasis at diagnosis in both unadjusted and age/sex-adjusted models (Table 3). However, the wide confidence intervals suggest considerable uncertainty in the effect size estimates and potential model instability. Similar results were also observed when differentiation grade was the outcome variable, with 2.5 times higher odds of presence of metastases at diagnosis in patients with poorly differentiated tumors (Table 6). In contrast, perihilar and distal tumor localizations were associated with a lower likelihood of poor differentiation with high variations when adjusted by sex (Table 6). Although some of the evaluated biomarkers showed statistical significance, the lower bounds of ORs close to the null value (one) indicate their limited clinical relevance. Furthermore, the wide confidence intervals around these ORs reflect considerable patient heterogeneity, model instability, and ultimately, inconclusive results for the broader population as expected in an exploratory study.

### 4.3. Strengths and Limitations of the Study

Our study brings substantial information on the Romanian population of CCA patients, with the largest demographic and clinical data, a previously under described population. Given the lower incidence of the disease in Europe overall and the conflicting previous results, there is a need for updated research in this specific group of patients. Diagnosis and management in CCA patients are highly time-sensitive because of their aggressive nature, and any new insight can improve clinical outcomes. Current guidelines recommend a definitive histopathological diagnosis or, in case of failure of diagnostic methods, to have a multidisciplinary decision for the diagnosis of CCA in suspicious cases [17]. Continuous updates of information on cholangiocarcinoma patients are essential due to the constantly changing characteristics of patients with this diagnosis and because of its aggressive nature.

Although the main strength of our study lies in the evaluation of a large-scale dataset in Romanian CCA patients, a series of limitations derived from study design and analysis of routinely collected data, and the study’s retrospective nature must be acknowledged. Targeting patients from a single hospital with a positive histopathological diagnosis using medical charts as a source of data, our study is susceptible to selection bias and unmeasured confounding. Data collection from medical charts is susceptible to incompleteness. The presence of missing data reduces the statistical power of our analysis and increases the uncertainty of our estimates, reflected in wide confidence intervals. In the absence of any treatment for missing data, the potential for residual bias is acknowledged, and the robustness of our findings should be interpreted accordingly. As an exploratory study, no multiple testing correction was applied. Thus, the risk of Type I errors is inflated, so the identified statistically significant results should be considered as preliminary results and must be validated in independent, confirmatory cohorts if considered relevant. Last but not least, our results must be interpreted beyond statistical significance. The wide confidence intervals and effect sizes near the null value indicate that our findings have limited clinical relevance, despite their statistical significance. While our study was not sufficiently powered to detect small effects, it provides valuable foundational data for future research.

## 5. Conclusions

In this Romanian cohort, we found that intrahepatic cholangiocarcinoma (CCA) was significantly associated with metastatic disease at diagnosis compared to other localizations of CCA. We also found that routinely collected lymphocyte-based ratios held limited value for the evaluation of patients with cholangiocarcinoma. This negative finding refines the utility of such biomarkers in CCA assessment. However, our results suggest a potential biological link between tumor aggressiveness and systemic inflammation that deserve further investigation in prospective studies, maybe considering dynamic measurements in the context of genetic and molecular tumor characteristics.

## Figures and Tables

**Table 1:** Derived lymphocyte-related ratios and other inflammation ratios.

Abbreviation-Name	Formula
NLR—Neutrophil-to-Lymphocyte Ratio	NLR = NEU/LYM
dNLR—derived Neutrophil-to-Lymphocyte Ratio	dNLR = NEU/(WBC−-NEU)
PLR—Platelet-to-Lymphocyte Ratio	PLR = PLT/LYM
LMR—n Lymphocyte-to-Monocytes Ratio	LMR = LYM/MON
MWR—Monocyte-to-White blood cell Ratio	MWR = MON/WBC × 00
N/LPR—Neutrophil-to-Lymphocyte and Platelet Ratio	N/LPR = (NEU × 100)/(LYM × PLT)
SIRI—Systemic Inflammatory Response Index	SIRI = (NEU × MON)/LYM
SII—Systemic Immune-inflammation Index	SII = (NEU × PLT)/LYM
AISI—Aggregate Index of Systemic Inflammation	AISI = (NEU × MON × PLT)/LYM

NEU—absolute neutrophil count; LYM—absolute lymphocyte count; WBC—absolute leukocyte count; PLT—absolute platelet count; MON—absolute monocyte count.

**Table 2:** Characteristics of patients stratified by metastases at diagnosis.

Variable	With Metastases n = 115	Without Metastases n = 212	*p*-Value
CCA type *			<0.001
intrahepatic (iCCA)	59 (51.3)	57 (26.9)	
perihilar (pCCA)	44 (38.3)	68 (32.1)	
distal (dCCA)	12 (10.4)	87 (41.0)	
Comorbidities *			
Diabetes Mellitus	31 (27)	51 (24.1)	0.563
Obesity	12 (10.4)	30 (14.2)	0.338
Hypertension	58 (50.4)	117 (55.2)	0.411
Cholangitis *	23 (20)	36 (17)	0.498
Cholelithiasis *	35 (30.4)	51 (24.1)	0.211
Choledocholithiasis *	8 (7.0)	14 (6.6)	0.903
CRP ^#^	1.9 [0.7 to 7.3], n = 100	0.9 [0.4 to 3.6], n = 158	<0.001
NLR ^#^	4.3 [3 to 7.1]	3.3 [2.3 to 5.1]	<0.001
dNLR ^#^	2.9 [2.1 to 3.8]	2.2 [1.6 to 3.1]	<0.001
PLR ^#^	178 [142 to 250]	157 [119 to 223]	0.029
LMR ^#^	2.4 [1.6 to 3.3]	2.8 [1.9 to 4]	0.022
N/LPR ^#^	1.5 [1.2 to 2.5]	1.2 [0.8 to 2]	0.004
MWR ^#^	7 [5.5 to 8.7]	7.3 [5.8 to 8.8]	<0.001
SIRI ^#^	2.7 [1.7 to 5.4]	1.8 [1.2 to 3.3]	0.369
SII ^#^	1192 [728 to 1987]	849 [535 to 1487]	<0.001
AISI ^#^	799 [378 to 1538]	510 [275 to 1039]	<0.001

* Data are reported as no. (%); Chi-squared test was used to test the association; ^#^ Data are reported as median [Q1 to Q3], where Q is the value of the quartile; n = sample size, when not reported, the metrics include all eligible patients; CRP—C reactive protein; NLR—Neutrophil-to-Lymphocyte Ratio; dNLR—derived Neutrophil-to-Lymphocyte Ratio; PLR—Platelet-to-Lymphocyte Ratio; LMR—Lymphocyte-to-Monocytes Ratio; MWR—Monocyte-to-White blood cell Ratio; N/LPR—Neutrophil-to-Lymphocyte and Platelet Ratio; SIRI—Systemic Inflammatory Response Index; SII—Systemic Immune-inflammation Index; AISI—Aggregate Index of Systemic Inflammation.

**Table 3:** Factors associated with the risk of metastases: crude and adjusted models for metastases at diagnosis.

Variable	Unadjusted Model			Adjusted Model		
	*p*-Value	AIC	OR [95% CI]	*p*-Value	AIC	OR [95% CI]
CCA type	<0.001	390		<0.001	391	
iCCA-dCCA			7.504 [3.709 to 15.185]			7.367 [3.636 to 14.925]
pCCA-dCCA			4.691 [2.3 to 9.568]			4.507 [2.202 to 9.227]
						1.323 [0.811 to 2.156] *
						0.983 [0.959 to 1.010] ^#^
NLR	<0.001	416	1.115 [1.042 to 1.192]			
dNLR	0.002	418	1.215 [1.069 to 1.380]			
PLR	0.005	420	1.003 [1.001 to 1.005]			
LMR	0.017	422	0.842 [0.732 to 0.978]	0.004	419	0.797 [0.679 to 0.935]
						1.648 [1.013 to 2.681] *
						0.980 [0.985 to 1.004] ^#^
N/LPR	0.007	421	1.181 [1.025 to 1.360]	0.004	419	1.223 [1.044 to 1.43]
						1.432 [0.863 to 2.30] *
						0.979 [0.956 to 1.00] ^#^
SIRI	<0.001	413	1.145 [1.060 to 1.236]	<0.001	410	1.162 [1.073 to 1.258]
						1.699 [1.051 to 2.748] *

*p*-value and AIC belong to the overall model test; * sex is a covariate with men as reference; ^#^ age at diagnosis is a covariate; iCCA—intrahepatic; pCCA—perihilar; dCCA—distal; NLR—Neutrophil-to-Lymphocyte Ratio; dNLR—derived Neutrophil-to-Lymphocyte Ratio; PLR—Platelet-to-Lymphocyte Ratio; LMR—Lymphocyte-to-Monocytes Ratio N/LPR—Neutrophil-to-Lymphocyte and Platelet Ratio; SIRI—Systemic Inflammatory Response Index.

**Table 4:** Patient’s characteristics stratified by tumor localization.

Variable	Intrahepatic (iCCA) n = 116	Perihilar (pCCA) n = 112	Distal (dCCA) n = 99	*p*-Value
Age, years	66 [60.8 to 70]	66 [58 to 72]	65 [59 to 72]	0.974
Comorbidities *				
Diabetes Mellitus	29 (25)	30 (26.8)	23 (23.2)	0.838
Obesity	15 (12.9)	17 (15.2)	10 (10.1)	0.546
Hypertension	63 (54.3)	59 (52.7)	53 (53.5)	0.970
Cholangitis *	6 (5.2)	33 (29.5)	20 (20.2)	0.465
Cholelithiasis *	26 (22.4)	33 (29.5)	27 (27.3)	0.042 ^&^
Choledocholithiasis *	6 (5.2)	4 (3.6)	12 (12.1)	0.903
CRP ^#^	1.84 [0.5 to 4.4], n = 85	1.37 [0.5 to 7.7], n = 94	0.97 [0.5 to 2.7], n = 79	0.142
NLR ^#^	3.65 [2.3 to 5.2]	3.65 [2.8 to 5.5]	3.5 [2.6 to 5.7]	0.439
dNLR ^#^	2.36 [1.6 to 3.1]	2.37 [2 to 3.4]	2.39 [1.8 to 3.4]	0.524
PLR ^#^	148 [117 to 200]	179 [132 to 260]	172 [137 to 233]	0.009
LMR ^#^	2.6 [1.8 to 3.9]	2.6 [1.6 to 3.9]	2.8 [2 to 3.9]	0.567
N/LPR ^#^	1.52 [0.9 to 2.2]	1.36 [0.9 to 2.2]	1.36 [0.9 to 2]	0.667
MWR ^#^	7.35 [6 to 8.8]	7.2 [5.4 to 8.7]	7 [5.8 to 8.8]	0.505
SIRI ^#^	1.95 [1.2 to 4.2]	2.37 [1.5 to 4.2]	1.91 [1.2 to 3.7]	0.315
SII ^#^	820 [553 to 1483]	1038 [667 to 1843]	1025 [572 to 1580]	0.065
AISI ^#^	524 [270 to 1202]	635 [378 to 1322]	550 [298 to 1152]	0.199

* Data are reported as no. (%); Chi-squared test was used to test the association; ^#^ Data are reported as median [Q1 to Q3], where Q is the value of the quartile and comparison was done with Kruskal–Wallis test; n = sample size, when not reported, the metrics include all eligible patients; ^&^ Fisher’s exact test. NLR—Neutrophil-to-Lymphocyte Ratio; dNLR—derived Neutrophil-to-Lymphocyte Ratio; PLR—Platelet-to-Lymphocyte Ratio; LMR—Lymphocyte-to-Monocytes Ratio; MWR—Monocyte-to-White blood cell Ratio; N/LPR—Neutrophil-to-Lymphocyte and Platelet Ratio; SIRI—Systemic Inflammatory Response Index; SII—Systemic Immune-inflammation Index; AISI—Aggregate Index of Systemic Inflammation.

**Table 5:** Characteristics stratified by differentiation grade.

Variable	G1-Well Differentiated n = 53	G2-Moderately Differentiated n = 97	G3-Poorly Differentiated n = 64	*p*-Value
Age, years ^#^	63 [58 to 70]	66 [58 to 70]	66 [62 to 71]	0.312
Sex, male *	29 (54.7)	58 (59.8)	39 (60.9)	0.769
Living place, rural *	31 (58.5)	69 (71.1)	39 (60.9)	0.217
CCA type *				0.048
intrahepatic (iCCA)	16 (17.2)	40 (43)	37 (39.8)	
perihilar (pCCA)	17 (28.8)	28 (47.5)	14 (23.7)	
distal (dCCA)	20 (32.3)	29 (46.8)	13 (21.0)	
Metastases at diagnosis *	13 (24.5)	30 (30.9)	34 (53.1)	0.002
Comorbidities *				
Diabetes Mellitus	9 (17)	25 (25.8)	18 (28.1)	0.358
Obesity	9 (17)	18 (18.6)	4 (6.3)	0.079
Hypertension	24 (45.3)	50 (51.5)	38 (59.4)	0.308
Cholangitis *	8 (15.1)	13 (13.4)	10 (15.6)	0.908 ^&^
Cholelithiasis *	8 (15.1)	24 (24.7)	16 (25)	0.337 ^&^
Choledocholithiasis *	4 (7.5)	4 (4.1)	6 (9.4)	0.403 ^&^
CRP ^#^	1.98 [0.5 to 7.8], n = 37	1.22 [0.5 to 3.5], n = 70	2.26 [0.6 to 6.9], n = 51	0.390
NLR ^#^	3.4 [2.9 to 5]	3.2 [2.4 to 5]	4.8 [2.9 to 6.7]	0.019
dNLR ^#^	2.42 [1.9 to 3]	2.27 [1.7 to 3.1]	3 [2.1 to 3.7]	0.036
PLR ^#^	145 [114 to 219]	152 [120 to 207]	181 [148 to 251]	0.012
LMR ^#^	2.8 [2.2 to 3.9]	2.9 [2 to 4]	2.3 [1.6 to 3.1]	0.015
N/LPR ^#^	1.57 [1 to 2.1]	1.31 [0.8 to 1.8]	1.66 [1 to 2.7]	0.025
MWR ^#^	6.5 [5.1 to 8.3]	7.3 [5.8 to 8.6]	7 [5.7 to 8.5]	0.303
SIRI ^#^	1.83 [1.2 to 3.5]	1.85 [1.2 to 3.9]	2.64 [1.4 to 5.2]	0.165
SII ^#^	856 [556 to 1263]	838 [595 to 1551]	1159 [737 to 1951]	0.089
AISI ^#^	541 [273 to 901]	514 [291 to 1134]	599 [330 to 1723]	0.223

* Data are reported as no. (%); Chi-squared test was used to test the association; ^#^ Data are reported as median [Q1 to Q3], where Q is the value of the quartile and comparison was done with Kruskal–Wallis test; n = sample size, when not reported, the metrics include all eligible patients; ^&^ Fisher’s exact test. NLR—Neutrophil-to-Lymphocyte Ratio; dNLR—derived Neutrophil-to-Lymphocyte Ratio; PLR—Platelet-to-Lymphocyte Ratio; LMR—Lymphocyte-to-Monocytes Ratio; MWR—Monocyte-to-White blood cell Ratio; N/LPR—Neutrophil-to-Lymphocyte and Platelet Ratio; SIRI—Systemic Inflammatory Response Index; SII—Systemic Immune-inflammation Index; AISI—Aggregate Index of Systemic Inflammation.

**Table 6:** Factors associated with the differentiation grade: crude and adjusted models.

Variable	Unadjusted Model			Adjusted Model		
	*p*-Value	AIC	OR [95% CI]	*p*-Value	AIC	OR [95% CI]
Metastases	<0.001	451	2.50 [1.46 to 4.32]			
CCA type	0.008	454		<0.001	449	
pCCA-iCCA			0.488 [0.262 to 0.901]			0.515 [0.275 to 0.956]
dCCA-iCCA			0.416 [0.224 to 0.764]			0.541 [0.284 to 1.024]
						2.186 [1.245 to 3.872] *
NLR	0.139	460	1.060 [0.983 to 1.140]			
dNLR	0.471	461	1.050 [0.919 to 1.210]			
PLR	0.022	457	1.000 [1.000 to 1.010]	<0.001	450	1.00 [1.00 to 1.00]
						2.29 [1.325 to 3.98] *
LMR	0.009	455	0.819 [0.701 to 0.951]	<0.001	449	0.855 [0.730 to 0.998]
						2.238 [1.298 to 3.899] *
N/LPR	0.269	461	1.10 [0.931 to 1.310]			

*p*-value and AIC belong to the overall model test; * metastases at diagnosis is a covariate; iCCA—intrahepatic; pCCA—perihilar; dCCA—distal. NLR—Neutrophil-to-Lymphocyte Ratio; dNLR—derived Neutrophil-to-Lymphocyte Ratio; PLR—Platelet-to-Lymphocyte Ratio; LMR—Lymphocyte-to-Monocytes Ratio.

## Data Availability

The raw data supporting the conclusions of this article will be made available by the authors on request.

## Abbreviations

The following abbreviations are used in this manuscript:

CCA	cholangiocarcinoma
iCCA	intrahepatic cholangiocarcinoma
eCCA	extrahepatic cholangiocarcinoma
pCCA	perihilar cholangiocarcinoma
dCCA	distal cholangiocarcinoma
OR	Odd Ratio
PSC	Primary Sclerosing Cholangitis
HBV	Hepatitis B virus
HCV	Hepatitis C virus
T2D	Type 2 Diabetes
ERCP	Endoscopic Retrograde Cholangiopancreatography
EUS	Endoscopic Ultrasound
FNB	Fine-Needle Biopsy
95% CI	95% confidence interval
CT	Computed Tomographic
MRCP	Magnetic Resonance Cholangiopancreatography
CA 19-9	Carbohydrate Antigen 19-9
CEA	Carcinoembryonic antigen
CA 125	Cancer Antigen 125
WBCs	White Blood Cells
WBC	Leukocyte
LYM	Lymphocytes
MON	Monocytes
NEU	Neutrophils
PLT	Platelets
CRP	C reactive protein
NLR	Neutrophil-to-Lymphocyte Ratio
dNLR	derived Neutrophil-to-Lymphocyte Ratio
PLR	Platelet-to-Lymphocyte Ratio
LMR	Lymphocyte-to-Monocytes Ratio
MWR	Monocyte-to-White blood cell Ratio
N/LPR	Neutrophil-to-Lymphocyte and Platelet Ratio
SIRI	Systemic Inflammatory Response Index
SII	Systemic Immune-inflammation Index
AISI	Aggregate Index of Systemic Inflammation
